# Comprehensive Small Animal Imaging Strategies on a Clinical 3 T Dedicated Head MR-Scanner; Adapted Methods and Sequence Protocols in CNS Pathologies

**DOI:** 10.1371/journal.pone.0016091

**Published:** 2011-02-07

**Authors:** Deepu R. Pillai, Robin M. Heidemann, Praveen Kumar, Nagesh Shanbhag, Titus Lanz, Michael S. Dittmar, Beatrice Sandner, Christoph P. Beier, Norbert Weidner, Mark W. Greenlee, Gerhard Schuierer, Ulrich Bogdahn, Felix Schlachetzki

**Affiliations:** 1 Department of Neurology, Regensburg University Medical Centre, Regensburg, Germany; 2 Department of Genetics and Neurobiology, Biozentrum, Julius-Maximilians-Universität Würzburg, Würzburg, Germany; 3 Department of Neurophysics, Max Planck Institute for Human Cognitive and Brain Sciences, Leipzig, Germany; 4 Siemens Healthcare Sector, Erlangen, Germany; 5 Department of Neurology, University Medical Centre, RWTH Aachen, Aachen, Germany; 6 RAPID Biomedical GmbH, Würzburg-Rimpar, Germany; 7 Department of Anaesthesiology, Regensburg University Medical Centre, Regensburg, Germany; 8 Institute for Paraplegia, University of Heidelberg, Heidelberg, Germany; 9 Institute for Experimental Psychology, University of Regensburg, Regensburg, Germany; 10 Center for Neuroradiology, Regensburg University Medical Centre and Bezirksklinikum Regensburg, Regensburg, Germany; Julius-Maximilians-Universität Würzburg, Germany

## Abstract

**Background:**

Small animal models of human diseases are an indispensable aspect of pre-clinical research. Being dynamic, most pathologies demand extensive longitudinal monitoring to understand disease mechanisms, drug efficacy and side effects. These considerations often demand the concomitant development of monitoring systems with sufficient temporal and spatial resolution.

**Methodology and Results:**

This study attempts to configure and optimize a clinical 3 Tesla magnetic resonance scanner to facilitate imaging of small animal central nervous system pathologies. The hardware of the scanner was complemented by a custom-built, 4-channel phased array coil system. Extensive modification of standard sequence protocols was carried out based on tissue relaxometric calculations. Proton density differences between the gray and white matter of the rodent spinal cord along with transverse relaxation due to magnetic susceptibility differences at the cortex and striatum of both rats and mice demonstrated statistically significant differences. The employed parallel imaging reconstruction algorithms had distinct properties dependent on the sequence type and in the presence of the contrast agent. The attempt to morphologically phenotype a normal healthy rat brain in multiple planes delineated a number of anatomical regions, and all the clinically relevant sequels following acute cerebral ischemia could be adequately characterized. Changes in blood-brain-barrier permeability following ischemia-reperfusion were also apparent at a later time. Typical characteristics of intra-cerebral haemorrhage at acute and chronic stages were also visualized up to one month. Two models of rodent spinal cord injury were adequately characterized and closely mimicked the results of histological studies. In the employed rodent animal handling system a mouse model of glioblastoma was also studied with unequivocal results.

**Conclusions:**

The implemented customizations including extensive sequence protocol modifications resulted in images of high diagnostic quality. These results prove that lack of dedicated animal scanners shouldn't discourage conventional small animal imaging studies.

## Introduction

Small animal models of human pathologies involving rats and mice are principal to further our understanding of various disease processes. They are also invaluable in the pre-clinical evaluation of potential drug candidates for their therapeutic efficacy and/or toxicity profiles. Pathologic states being dynamic in nature makes it mandatory to monitor them in a longitudinal fashion. This necessitates the use of reliable non-invasive monitoring techniques, which, should satisfy a minimal set of criteria like, 1) adequate spatial resolution and signal-to-noise ratio (SNR), 2) ability to morphologically differentiate tissues with required contrast, along with 3) a good temporal resolution[1].

Magnetic resonance imaging (MRI) provides excellent soft tissue contrast coupled to a good temporal and spatial resolution, making it an indispensable tool to discriminate between normal and pathologically altered tissues for small animal studies, particularly, that of rodents[2]. Moreover, the spatial resolution is further aided by the spectral nature of MRI where, the size of the details resolved can be less than the wavelength of the radiation involved[3].

Recently, MRI has turned out to be much more than an indispensable non-invasive routine diagnostic imaging modality in clinical routine and is now finding increasing applications in pre-clinical research [4], [5], [6], [7]. However, it cannot be emphasized enough that, MR imaging strategies of small animals like that of a mouse poses unique technical challenges as a 4,000 times weaker MR signal should be measured in a mouse (weight: 25 g) compared to that measured in a 100 kg man. If identical hardware is used the spatial resolution needs to be scaled relative to the anatomy, so that a typical clinical voxel size of 10 mm^3^ scales down to 0.0025 mm^3^ in a mouse[8].

To address such concerns, purpose dedicated small animal MR scanners with field strengths ranging from 4–21 Tesla(T) have been developed and are commercially available enabling full range of applications including MR spectroscopy and functional MRI in small animals[9], [10], [11], [12]. Though impressive, such a dedicated small animal imaging facility is resource intensive and calls for a wide array of expert personnel and long term commitment which is just beyond the scope of many research laboratories.

The rapid increase in signal scaling with field strength is the only factor contributing to improved image quality at high fields. All other factors, in fact, work against higher field strengths except in cases of, functional and spectroscopic MR studies. In conventional imaging, the penalties for increased field strength include increased noise, susceptibility and chemical shift artefacts, significantly higher radio frequency (RF) power deposition and tissue heating, reduced longitudinal relaxation (T_1_) contrast, inhomogeneity effects due to eddy currents and wavelength effects[13]. In practice, to maintain the chemical shift effect at a constant number of pixels, the frequency encoding bandwidth (BW) has to increase in proportion to B_0_, thus reducing the gain in SNR. Further, once decreasing tissue conductivity and increasing T_1_ are taken into account, it could be argued that the real gain in SNR is even lower.

Meanwhile, a number of groups have performed small animal structural, spectroscopic and functional studies on the widely available, relatively low-field clinical scanners with modest success. Most of these studies have been carried out at the rather widely available 1.5 T clinical scanners [14], [15], [16]. Higher field clinical MR scanners at 3 T are now being increasingly deployed, but their utility in small animal imaging studies pales in comparison to studies at 1.5 T even though such attempts are well in progress [17], [18], [19], [20]. Another sub-class of these 3.0 T clinical scanners belong to that of dedicated head scanners. As they are primarily intended to perform ultra-fast functional MRI in humans, their design features include significantly shorter magnets and narrower bores compared to their whole body counterparts and are therefore better suited for investigations of smaller objects [21].

The present study aims to detail the methodologies and customizations followed in configuring not just a clinical whole body MRI system, but that, on a relatively rarer dedicated-head scanner at 3 T for studying pathologies involving rat models of a) ischemic and hemorrhagic stroke, b) spinal cord lesions following wire knife cut and contusion injury at the cervical and thoracic level respectively and, c) a mouse model of glioblastoma (GBM). The obtained results clearly demonstrate the benefits brought about by customized hardware, in synergy with purpose directed sequence protocol modifications could yield conventional images of high diagnostic quality.

## Materials and Methods

### Ethics Statement

All animal experiments were carried out in accordance with European communities council directive (86/609/EEC) and institutional guidelines for animal care after local ethics committee approval (Ethics committee for animal laboratories, Medical Faculty, University of Regensburg, 93042, Regensburg, Germany). The human cancer stem cell (HCSC) induced orthotopic xenograft mouse model of GBM was developed after approval by the local authorities governing health care (Regierung der Oberpfalz, Emmeransplatz 8, 93047, Regensburg, Germany, www.ropf.de; AZ: 54-2531.2-22/08).

### 1. The MR system

The employed clinical MR system is the US-FDA approved, high field, 2.895 Tesla dedicated head MR scanner (Siemens Magnetom Allegra, Siemens Healthcare Sector, Erlangen, Germany). The incorporated gradient sub-system achieves a peak amplitude of 40 mT/m with a slew rate of 400 mT/m/ms. The RF system comprises of a fully digital transmit-receive compact solid state RF amplifier at 123.2 MHz with a receiver BW of 1 MHz.

### 2. The Phased Array Radiofrequency Coil system

To facilitate small animal imaging, MR system hardware was complemented by a 4-channel rat brain and spine phased array coil assemblies. The RF assembly was complete with an animal handling system with provisions for inhalational anaesthetic supply and a feedback regulated heating pad (RAPID Biomedical, Rimpar, Germany). In order to ensure uniform excitation across the region of interest (RoI), these receive-only coils were combined to a volume resonator (RAPID Biomedical, Rimpar, Germany) with an inner diameter of 69 mm.

**Figure 1:**
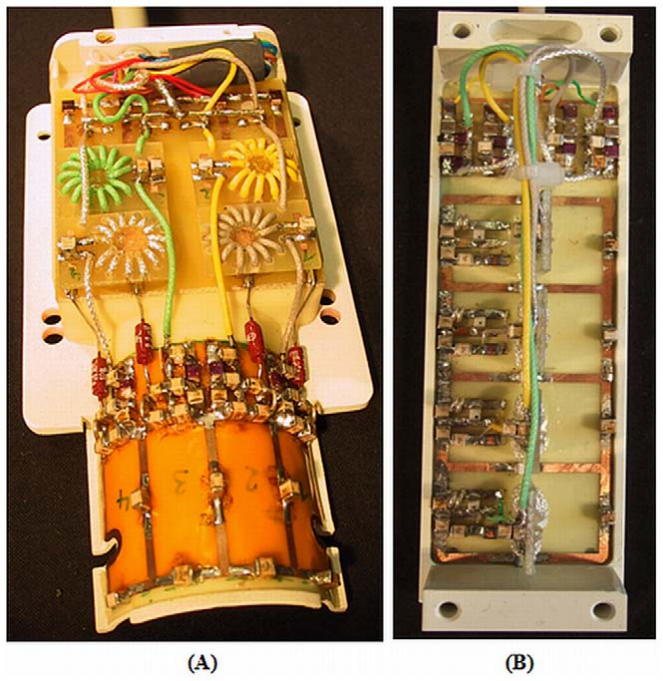
Geometry of the 4-channel phased array radio frequency coil system. (A) Rat head coil array where the 4 coil elements are placed in a half-cylindrical design along the X-axis of the scanner. (B) Rat spine coil array where the 4 coil elements are placed along the length of the Z-axis of the scanner.

The rat head receive-only RF coil array is fixed tuned to the operating frequency of 123.2 MHz. The four rectangular coil elements were aligned along the X-axis covering an area of 46 mm (Length)×31 mm (Width) placed in a half-cylindrical design to accommodate the rat head (Figure 1A). The overall size of the array is chosen to cover the entire rat brain. The coil elements have a ‘Q’ value of 110, which means a ‘Q’ drop by a factor of 1.15, when compared to the unloaded coil. The impedance of all elements is fixed tuned and matched to 50 Ω when loaded with a rat head. Active decoupling of the coil elements during RF transmission is achieved with trap circuits switched by PIN diodes, found to be better than 30 dB. Mutual coupling of neighbouring elements is compensated by a shared inductor design. Preamplifier decoupling (better than 20 dB, where applicable, added up to the decoupling of next neighbours) ensures decoupling of all the other element paizrs.

The rat spine receive-only RF coil array is again fixed tuned, matched and is combined with the same volume transmit coil. Here the four array elements are aligned along the Z-axis to form a rectangle of dimensions 80 mm (Length)×30 mm (Width) (Figure 1B). The unloaded ‘Q’ of each coil element was 144, dropping by a factor of 2, when loaded. In the spine array, neighbouring elements are decoupled by an overlap design. Again, active decoupling by trap circuits (better than 35 dB) and preamplifier decoupling are used for achieving decoupling from the transmit coil and between pairs of coil elements.

### 3. In-Vivo MRI

#### 3.1. Configuration and optimization of sequence parameters

All implemented sequence parameters are modified from the pre-loaded standard Siemens product sequences. A number of modifications pertaining to spin-echo (SE), turbo spin-echo (TSE), gradient recalled echo (GRE), diffusion weighted and perfusion echo planar imaging (DW- & PE-EPI) sequences have been addressed. All modifications are aimed to ensure maximum signal with enough in-plane resolution for sensitive detection and spatial allocation of lesions by eliminating artefacts. In our approach, acquisition times (AT) were given the least priority and were traded against factors contributing to enhanced image quality.

In the 2-dimensional mode, all acquisitions, irrespective of the sequence type has been performed in multi-slice interleaved fashion to enhance signal by eliminating slice crosstalk. Slice thickness (ST) of the rodent brain was maintained at 1 mm in the case of SE, TSE and EPI sequences, primarily to facilitate cross comparison with histological tissue sections prepared by the rodent brain matrix (ASI Instruments, Inc. USA) where the minimum ST is limited to 1 mm. However, due to gradient strength limitations, the minimum achievable ST with GRE sequences was 1.5 mm. The same ST (1.5 mm) was considered for the rodent spine primarily to enhance the available signal due to the small size of the object of interest. Gradient performance also imposed limits on minimizing the field of view (FoV) which, was confined to 50 mm in the case of EPI and GRE sequences, which, could be further reduced to 25 mm in the case of SE and TSE sequences. However, in the case of rodents a 25 mm FoV, resulted in anatomical regions extending beyond the RoI causing aliasing artefacts along the phase encode direction and were countered by employing phase oversampling.

Further, the phase encoding direction was always kept maintained in the right-left direction (X-axis of the scanner) as the phase encoding along the anterior-posterior direction (Y-axis of the scanner) demonstrated motion related artefacts. The only exception to this case was that with DW-EPI sequence, because, such a scheme resulted in relatively higher geometric distortions along the phase encode direction. In order to maximize the available signal and to reduce noise, minimal receiver BW had been resorted to wherever possible as in the case with SE and GRE sequences. However, the BW selection for T_2_- and PD-TSE sequences was guided by the tissue T_2_ value and the corresponding echo-spacing resulting from the selected echo train length (Turbo factor).

EPI, particularly at higher field strengths are prone to geometric distortions, signal loss and image blurring caused by frequency shifts and T_2_^*^ relaxation distortion of the MR signal along the k-space trajectory due to magnetic field inhomogeneities[22]. A two-tier strategy has been adopted to minimize such distortions involving, 1) manual shimming of the magnet has been carried out following an auto-shimming routine to achieve a proton BW ≤35 Hz at full width half maximum with an accompanying T_2_^*^ value ≥30. 2) Due to the availability of multiple receiver channels, parallel imaging (PI) has been implemented for all EPI sequences[23]. The k-space based reconstruction algorithm (RA) allowed by generalized auto-calibrating partially parallel acquisition (GRAPPA) at an acceleration factor (AF) of 3 was considered for DW-EPI and PE-EPI was performed at an AF of 4. Further, for EPI sequences the FoV in the phase encoding direction was maintained at or less than 50% of the FoV in read direction as this reduces the acquisition time in case of DW-EPI and contributes to enhanced temporal resolution of the PE-EPI sequence. 

In our lab, GRE sequences are primarily considered to detect haemorrhage, haemorrhagic streaks and haemosiderin deposits in the brain with maximum possible sensitivity. However, susceptibility artefacts arising out of magnetic field in-homogeneities were minimized by employing 1) manual magnet shimming, 2) low time-to-echo (TE) value, and 3) a higher acquisition matrix[24].

Optimization of sequence parameters can never be considered adequate without determining the tissue relaxation characteristics, which in turn decides the time-to-repeat (TR) and time-to-echo (TE) values. Currently, there are no quantitative reports on the basic MR properties of the rat brain, spine and mouse brain at 3 T in the literature. Knowledge of these properties is essential for determining the mechanisms that are responsible for the endogenous MRI contrast between the gray and white matter (GM & WM) of the spinal cord, establishing sensitivity and specificity of the MR parameters to pathological changes seen in normal and injured tissues, and describing the signal enhancement features achieved with the use of exogenous contrast agents.

Therefore, the first aim of this work was to perform baseline measurements of the spin-lattice (T_1_), spin-spin (T_2_), relaxation times at 3 T for the rat and mouse brains along with determination of the relative proton density (rel-PD) values of the gray and white matter of the rat spine. Further, T_2_^*^ relaxation due to magnetic susceptibility effects has also been derived for the rat and mouse brains. Moreover, since two PI RAs like modified sensitivity encoding (mSENSE) and GRAPPA were available to choose from for DW- and PE-EPI sequences, these strategies has been compared and contrasted against each other primarily for the available SNR and also for any gross geometric distortions and reconstruction related artefacts.

#### 3.2. Determination of tissue relaxation characteristics

Male Wistar rats (N = 8) (Charles River Laboratories, Sulzfeld, Germany) weighing 250–300 g and male in-house bred NMRI^(nu/nu)^ mice (N = 8) weighing 20–25 g were used for the determination of the brain cortical and striatal T_1_, T_2_ and T_2_^*^ relaxation characteristics.

Anaesthesia was induced using 5% Isoflurane for rats and mice and the anaesthetic state was maintained with 1.5 and 0.5% Isoflurane respectively. Animals were mounted in prone position within the scanner and the body temperature was kept maintained with feedback regulated heating pad. Normal spine gray and white matter has been characterized on female Fischer 344 rats (N = 8) (Charles River Laboratories, Sulzfeld, Germany) weighing 160–180 g. Following anaesthesia animals were mounted in supine position over the rectangular spine coil array housing and the parameters determined also include rel-PD values along with T_1_ and T_2_ profile. The parameter dataset for characterizing these tissue relaxation values are detailed in Table 1.

**Table 1:** Parameter dataset employed for calculating tissue relaxation characteristics.

T_1_-Relaxation (IR)			T_2_-Relaxation (SE)		T_2_^*^-Relaxation (GRE)	
TR(ms)	TE(ms)	IT(ms)	TR(ms)	TE(ms)	TR(ms)	TE(ms)
10,000	74	500	4000	29	700	20
		800		58		30
		1000		88		40
		1500		117		50
		2000		146		60
		4000		204		
		6000				
		8000				

IR, inversion recovery; SE, spin-echo; GRE, gradient recalled echo; TR, repetition time; TE, echo time; IT, inversion time.

#### 3.3. Determination of the optimal parallel imaging reconstruction algorithm: GRAPPA or mSENSE?

The criteria chosen for the selection of the optimal PI strategy was based on the available SNR and also on the absence of any gross geometric distortions. SNR calculations were carried out by the “difference method” referred hereafter as SNR_diff_ based on the evaluation of a difference image from two repeated (identical) acquisitions as described previously[25]. Two separate, but, identical datasets for each of the DW- and PE-EPI were acquired from an anesthetized male Wistar rat weighing 250–300 g employing both GRAPPA and mSENSE. In the case of DW-EPI, acquisitions were performed at all the possible AFs (from 2–4) and an image set without PI was also acquired for the sake of comparison. PE-EPI acquisitions were carried out without any contrast agent administration and were performed at AFs of 3 and 4 as the AF of 2 and acquisition without PI prolonged the TR value and affected the temporal resolution of the PE-EPI sequence. All the other EPI sequence parameters employed are as described in Table 2.

**Table 2:** Modified sequence parameters employed for facilitating small animal imaging.

Pathology	Ischemic stroke				ICH	Spine Injury	GBM	
Animal	Rat						Mouse	
Sequence	DW -EPI	PE-EPI	T_2_-TSE	T_1_-SE	T_2_ *-GRE	PD + T_2_-TSE	T_2_-TSE	T_1_-SE
**TR (ms)**	3000	900	3000	900	500	5000∼7000*	4000	1000
**TE (ms)**	90	27	70	10	20	13, 80	68	18
**FoV (Read,cm)**	5.7	5.7	2.5	2.5	5.0	2.5	2.5	2.5
**FoV (Phase, %)**	46.9	50	100	100	100	100	100	68.8
**IM**	128×60	64×128	128×128	128×128	256×256	128×128	128×128	88×128
**ISG (%)**	0	0	0	0	0	0	0	0
**FA(°)**	-NA-	130	180	130	20	180	180	90
**BW(Hz/Pixel)**	752	752	94	158	30	150	40	90
**PED**	A-P	R-L	R-L	R-L	R-L	R-L	R-L	R-L
**POS (%)**	0	50	50	50	0	100	0	0
**TF/EPI factor**	60	64	7	-	-	5	5	-
**ES(ms)**	1.52	1.54	23.2	-	-	13.3	34.2	-
**MSM**	IL	IL	IL	IL	IL	IL	IL	IL
**Series**	IL	IL	IL	IL	IL	IL	IL	IL
**NoA**	4	2	4	3	2	4	4	4
**PI(GRAPPA)**	Yes	Yes	No	No	No	No	No	No
**AF**	3	4	-	-	-	-	-	-
**NoM**	-	50	-	-	-	-	-	-
**DD**	3	-	-	-	-	-	-	-
**‘b’ values(s/mm^2^)**	0	-	-	-	-	-	-	-
	500	-	-	-	-	-	-	-
	1000	-	-	-	-	-	-	-
	1500	-	-	-	-	-	-	-
	2000	-	-	-	-	-	-	-
	2500							

ICH, intra-cerebral haemorrhage; GBM, glioblastoma; DW-EPI, diffusion weighted echo planar imaging; PE-EPI, perfusion echo planar imaging; T_2_-TSE, T_2_-weighted turbo spin echo; T_1_-SE, T_1_-weighted spin echo; T_2_^*^-GRE, susceptibility weighted gradient recalled echo; PD-TSE, proton density weighted turbo spin echo; TR, repetition time; TE, echo time; FoV, field of view; IM, image matrix; ISG, inter slice gap; FA, flip angle; BW, band width; PED, phase encoding direction; POS, phase over-sampling; TF, turbo factor; ES, echo spacing; MSM, multi-slice mode; NoA, number of averages; PI, parallel imaging; GRAPPA, generalized auto-calibrating partially parallel acquisition; AF, acceleration factor; NoM, number of measurements; DD, diffusion direction; A-P, anterior-posterior; R-L, right-left; IL, interleaved.

*Depending upon the respiration rate of the animal.

#### 3.4. Anatomical characterization of a rat brain

As a prelude to pathological rat brain imaging, an attempt has been made for phenotypic characterization of the rat brain using a 3-Dimensional T_1_-weighted magnetization prepared rapid acquisition gradient echo (3D-MPRAGE) sequence. This was performed on a healthy male Wistar rat weighing 250–300 g. The animal was anaesthetized and positioned within the scanner as mentioned before. After acquiring localizer images, 3D-MPRAGE acquisition was performed with TR  = 1070 ms, TE  = 4.95 ms, Inversion time (TI)  = 900 ms, FoV  = 50 mm, Image matrix (IM)  = 256×256 and BW = 130 Hz/Px.

#### 3.5. MR imaging of a rat model of ischemic stroke

Three male Wistar rats weighing 250–300 g were considered for this study. Following anaesthesia induction, the animals were endotracheally intubated and mechanically ventilated (RS Biomed ventilator, Sinzing, Germany) with 1.5% Isoflurane in 30%:70% oxygen:nitrous oxide mixture. The femoral vein was exposed and cannulated for contrast agent injection. Transient middle cerebral artery occlusion (tMCAO) was then performed for inducing cerebral ischemia as described by Longa et al. with modifications by Spratt et al.[26], [27]. One hour following tMCAO, animals were anaesthetized as mentioned before, mounted on the animal holder of the scanner and the body temperature was kept maintained. After acquiring localizer images, DW-EPI acquisitions with trace weighted apparent diffusion co-efficient (ADC) maps were generated to confirm ischemic injury followed by T_2_-weighted turbo spin-echo (T_2_-TSE) acquisition. Cerebral blood perfusion characteristics were determined with PE-EPI sequences. Two PE-EPI acquisitions were attempted employing both mSENSE and GRAPPA at an AF of 4 in a time interval of 25∼30 minutes. PE-EPI with mSENSE was performed first and after 25 minutes another PE-EPI acquisition was carried out with GRAPPA at the same AF. For both PE-EPI acquisitions, first 3 measurements were ignored and following another five baseline measurements, Gadolinium diethylenetriamine-penta-acetic acid (Gd-DTPA, 0.2 mmol/kg, Magnevist®, Shering, Germany) was injected through the femoral vein within a sub-second time duration without saline flush. Relative cerebral blood perfusion characteristics like blood flow (rel-CBF) and blood volume (rel-CBV) maps were also derived from the acquired perfusion images. In another animal, following 90 minutes of tMCAO, the animal was reperfused for four hours. After acquiring all the parameters except that of PE-EPI as in the above mentioned case, the integrity of the blood brain barrier (BBB) was evaluated by a post-contrast T_1_-SE sequence following 25 minutes of Gd-DTPA administration. Modified sequence parameters employed are as detailed in Table 2.

#### 3.6. MR imaging of a rat model of intra-cerebral haemorrhage

Male Wistar rats (N = 2) weighing 250–300 g were subjected to the same anaesthetizing regimen as in the case of ischemic stroke. The rat model of intra-cerebral haemorrhage (ICH) was prepared as described previously [28]. Within one hour of surgery, the animals were positioned in the scanner as mentioned above. T_2_-TSE and T_2_^*^-GRE images were acquired following localizer scans. T_2_-TSE sequence parameters were identical to those used for cerebral ischemia whereas, modified parameters for T_2_^*^-GRE acquisition were used as in Table 2. The present study was conducted longitudinally with multiple imaging time points at <1, 72 hours, 1 week and 1 month post injury.

#### 3.7. MR imaging of rat models of spinal injuries

MR imaging of rodent spine was first attempted at the thoracic (T10) level on a normal healthy adult female Fischer rat weighing 160–180 g. Anaesthesia was induced using 5% Isoflurane - air mixture, following which, the animal was placed supine, over the rectangular surface of the rodent spine array coil housing. Anaesthesia was continued with 1.5% Isoflurane and the animal was carefully observed for a variable time period (10–15 minutes) to ensure rhythmic and automatic breathing, before, the MR scanner was synchronized to the respiratory cycle (ECG Trigger Unit HR V02, RAPID Biomedical, Rimpar, Germany) by placing the button sized air-cushion sensor below the diaphragm. A trigger delay of varying time interval (200–300 ms) was introduced to obtain motion-artefact free images.

Following satisfactory results, spine imaging was further extended to two models of spinal cord injuries. The first model was based on a Tungsten wire knife induced cut at the rostral-cervical (C3) spinal cord and the second model was based on spine contusion injury induced at the thoracic (T10) level in adult female Fischer rats weighing 160–180 g as described[11], [29]. Three animals were included in the first model whereas, five animals were considered for the second model. MR imaging was performed at 30 days post-injury with the aim of studying long-term MRI signatures of spinal injury except for one animal with the cervical cut studied at 24 hours post injury. Animals with cervical and contusion injuries were mounted in prone and supine positions respectively, and all the other settings were as in the case of normal spine image acquisition as mentioned above. Cervical and thoracic spine was characterized using modified T_2_ and PD - TSE sequences, the details of which are as provided in Table 2. Following this procedure, animals were immediately sacrificed and spine tissue was processed for Nissl and Prussian blue staining as described elsewhere[11].

#### 3.8. MR imaging of a mouse model of glioblastoma

The human cancer stem cell (HCSC) induced orthotopic xenograft mouse model of GBM was developed as described [30]. Three months after HCSC injection, animals (N = 15) were anesthetized with 0.5% Isoflurane and T_2_-TSE and an identical T_2_^*^-GRE sequence as in the case of the rat model of ICH were primarily considered to characterize the evolving tumour and haemorrhage if any, respectively. To assess BBB and/or blood tumour barrier (BTB) permeability characteristics, Gd-DTPA (0.2 mmol/kg) was administered by cardiac puncture. Post-contrast T_1_-SE images were acquired after 25 minutes. All sequence parameters are as detailed in Table 2. The brains were immediately removed; frozen and 10-micrometer sections were stained with Hematoxylin and Eosin. The degree of correlation between the tumour volumes obtained from the T_2_-weighted sequence has also been compared to those obtained by histological studies.

### Data Analysis

Mono-exponential non-linear curve fitting for performing quantitative relaxometry and two-tailed un-paired ‘t’ tests for comparing the relaxometric values of the cortex and striatum of the rat and mouse brains and comparisons of the gray and white matter of the spinal cord along with the determination of Pearson correlation co-efficient between the mouse tumour volumes obtained from T_2_-TSE images and histological studies were performed using Graphpad Prism Version 5.00 for Windows (Graphpad Software, San Diego, California, USA).

For calculating SNR_diff_ with DW-EPI images, identical RoIs were defined over the sub-cortical regions over a 0.7 sq.cm circular area on a pair of 1 mm thick slices (acquired as two identical separate set of images) located 6 mm posterior to the frontal cortex employing the built-in image analysis tools of the Siemens *Syngo*MR 2004A platform (Siemens Healthcare Sector, Erlangen, Germany). In the case of PE-EPI images, RoIs were defined on the cortical region for the same area and slice position as in the case of DW-EPI images. The resultant values of signal intensity and their differences in standard deviation (SD) are substituted in the following equation to obtain the SNR as described elsewhere[25].
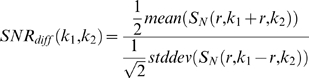
Where, ‘k_1_’ and ‘k_2_’ represent the two identical image acquisitions at identical RoIs as represented by ‘r’.

Cerebral blood perfusion parameters including rel-CBV and -CBF maps were derived by defining an RoI at the contralateral hemisphere covering the origin of the right MCA to allow measurement of the arterial input function, from which pixels representative of the right MCA branch were selected. Following this, perfusion maps were derived automatically with the built-in *Syngo*MR 2004A software. Regions of altered BBB permeability to Gd-DTPA were located by generating subtraction maps from pre- and post-contrast T_1_-SE images using the same built-in software tools.

## Results

### 1. Configuration and optimization of sequence parameters

The T_1_, T_2_ and T_2_^*^ values determined at the cortical and striatal regions of the rat and mouse brains are given in Table 3. All these values exhibited negligible inter-hemispherical differences. Rat spinal relaxation values (T_1_ and T_2_) along with rel-PD values at the gray and white matter of the thoracic (T10) region are also detailed. Statistically significant differences were found only between T_2_^*^ values of cortex and striatum of the rat and mouse brains and rel-PD values of gray and white matter of rat spine.

**Table 3:** Tissue relaxation characteristics at the considered anatomical regions of the rodent brain, spinal cord and mouse brain.

Animal	Rat				Mouse	
Brain/Spine	Brain (Mean ± SEM)		Spine (Mean ± SEM)		Brain (Mean ± SEM)	
Region	Cortex	Striatum	Gray matter	White matter	Cortex	Striatum
**T_2_-value (ms)**	76.69±3.9	69.995±3.9	79.92±4.97	87.65±9.32	77.8±13.1	69.29±6.74
**T_1_-value (ms)**	1064.76±18.45	968.64±31.9	730.8±70.62	871.65±91.22	1201.5±70.1	1081.5±56.73
T_2_^*^-value (ms)^*^	67.71±4.169	53.17±4.4	-	-	32.13±5.97	20.39±2.92
rel-PD (%)^*^	-	-	56.02±0.96	43.98±0.96	-	-

T_2_-value, transverse relaxation time; T_1_-value, longitudinal relaxation time; rel-PD, relative proton density, *P<0.0001, considered extremely significant. T_2_*-value, susceptibility weighted transverse relaxation time, *P = 0.0254 (Rat), 0.0185 (Mouse), considered significant.

The PI RAs, GRAPPA and mSENSE demonstrated distinctly different properties in relation to mean signal, their SD_diff_ and reconstruction related artefacts for the employed EPI sequences. Irrespective of DW- or PE-EPI sequences, implementation of PI consistently shortened the TE value and thereby demonstrated an increase in mean signal. In the case of DW-EPI, GRAPPA reconstruction at an AF of 3 demonstrated peak SNR accompanied by minimal SD_diff_, whereas, parallel acquisition with the maximum allowed AF of 4 resulted in reconstruction related artefacts. DW-EPI with mSENSE RA at an AF of 4 could not be completed as the image reconstruction programme raised exceptions. Further, mSENSE RA provided lower mean signal intensity and increased SD_diff_ compared to that of GRAPPA with AFs of 2 & 3. In the case of PE-EPI, mSENSE acquisitions demonstrated higher mean signal intensity and lower variations in SD_diff_ at the AFs of 3 and 4 compared to that of GRAPPA based RA.

**Figure 2:**
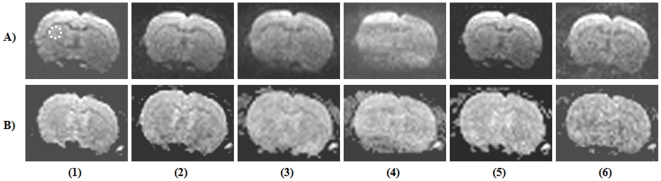
Diffusion weighted echo planar images (DW-EPI) of a rat brain with and without parallel imaging. Row (A) DW-EPI images (b = 1000 s/mm^2^) and row (B) their corresponding apparent diffusion co-efficient (ADC) maps. First column contains images acquired without implementing parallel imaging. Columns (2–4) contains image sets acquired with the reconstruction algorithm (RA), generalized auto-calibrating partially parallel acquisition (GRAPPA) employing acceleration factors (AFs) from 2–4. Columns (5–6) represent images acquired with the RA, modified sensitivity encoding (mSENSE) at AFs 2 & 3. The white dotted circle represents the area considered for calculating the signal-to-noise ratio.

**Figure 3:**
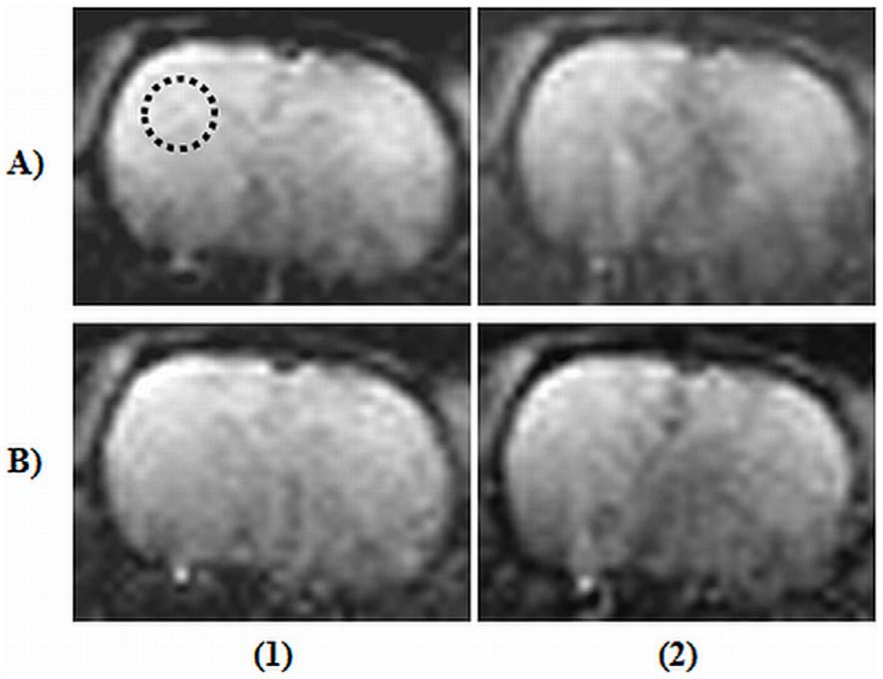
Perfusion echo planar images (PE-EPI) of a rat brain with parallel imaging. Two reconstruction algorithms namely, generalized auto-calibrating partially parallel acquisition (GRAPPA) and modified sensitivity encoding (mSENSE) at acceleration factors (AFs) 3 & 4 has been considered. Image sets (A1) and (A2) represents PE-EPI images acquired with GRAPPA at AFs 3 & 4 respectively and images (B1) and (B2) includes acquisitions with mSENSE at the same AFs. The black dotted circle represents the area considered for calculating signal-to-noise ratio.

The change in mean signal intensity, SD_diff_, and the calculated SNR for both GRAPPA and mSENSE at the considered AFs are detailed in Table 4 & 5 for DW- and PE-EPI respectively. Representative DW-EPI images (b = 1000 s/mm^2^) acquired with both GRAPPA and mSENSE along with their corresponding ADC maps are shown in Figure 2. Further, PE-EPI acquisitions with both RAs acquired with the two AFs are shown in Figure 3. The acquired image characteristics are as detailed in Table 6.

**Table 4:** Effect of two reconstruction algorithms on the signal- to- noise ratio with diffusion weighted echo planar imaging.

RA	PI features				Image set-01			Image set-02				
	AF	TE		ACS	SI	SD		SI	SD	MI	SD_diff_	SNR
**GRAPPA**	4		87	45	130.2	15.7		129	19.1	129.6	3.4	26.95
**mSENSE**	4		87	45	Image reconstruction failed!							
**GRAPPA**	3		90	39	116.4	11.1		114.6	10.8	115.5	0.3	272.24
**mSENSE**	3		90	39	113.3	15.1		112	18.2	112.65	3.1	25.7
**GRAPPA**	2		99	30	93.8		14.9	93.1	14	93.45	0.9	73.42
**mSENSE**	2		99	30	90.8		16.9	97.6	15.8	94.2	1.1	60.55
**No RA**	–		128	–	64.6		11.4	65.4	10.8	65	0.6	76.6

RA, reconstruction algorithm; PI, parallel imaging; AF, acceleration factor; TE, echo time; ACS, auto-calibration scanning lines; SI, signal intensity; SD, standard deviation; MI, mean intensity; SD_diff_, standard deviation difference; SNR, signal-to-noise ratio, GRAPPA, generalized auto-calibrating partially parallel acquisition; mSENSE, modified sensitivity encoding.

**Table 5:** Effect of two reconstruction algorithms on the signal- to- noise ratio with perfusion echo planar imaging.

RA	PI features				Image set-01		Image set-02				
	AF	TE		ACS	SI	SD	SI	SD	MI	SD_diff_	SNR
**GRAPPA**	4		27	70	416.7	42.4	430.8	39.5	423.75	2.9	103.32
**mSENSE**	4		27	70	498.4	52.5	492	53.3	495.2	0.8	437.7
**GRAPPA**	3		32	63	387.9	21.5	375.1	18	381.5	3.5	77.07
**mSENSE**	3		32	63	411.6	34.7	397.9	33	404.75	1.7	168.35

RA, reconstruction algorithm; PI, parallel imaging; AF, acceleration factor; TE, echo time; ACS, auto-calibration scanning lines; SI, signal intensity; SD, standard deviation; MI, mean intensity; SD_diff_, standard deviation difference; SNR, signal-to-noise ratio, GRAPPA, generalized auto-calibrating partially parallel acquisition; mSENSE, modified sensitivity encoding.

**Table 6:** Image acquisition characteristics of the different employed sequence types.

Pathology	Ischemic stroke				ICH	Spine Injury	GBM	
Animal	Rat						Mouse	
Sequence	DW-EPI	PE-EPI	T_2_-TSE	T_1_-SE	T_2_^*^-GRE	T_2_+ PD TSE	T_2_-TSE	T_1_-SE
**IPR(**µ**m)**	0.4×0.4	0.4×0.4	0.2×0.2	0.2×0.2	0.2×0.2	0.2×0.2	0.2×0.2	0.2×0.2
**ST(mm)**	1	1	1	1	1.5	1.5	1	1
**NoS**	15	15	15	15	10	20	9	9
**AT(min:sec)**	3.36	1:40	5:39	11:34	4:18	20:00∼25:00^*^	10:28	11:48

ICH, intra-cerebral haemorrhage; GBM, glioblastoma; DW-EPI, diffusion weighted echo planar imaging; PE-EPI, perfusion echo planar imaging; T_2_-TSE, T_2_-weighted turbo spin echo; T_1_-SE, T_1_-weighted spin echo; T_2_*-GRE, susceptibility weighted gradient recalled echo; PD-TSE, proton density weighted turbo spin echo; IPR, in-plane resolution; ST, slice thickness; NoS, number of Slices; AT, acquisition time.

### 2. 3D-MPRAGE characterization of rat brain anatomy

**Figure 4:**
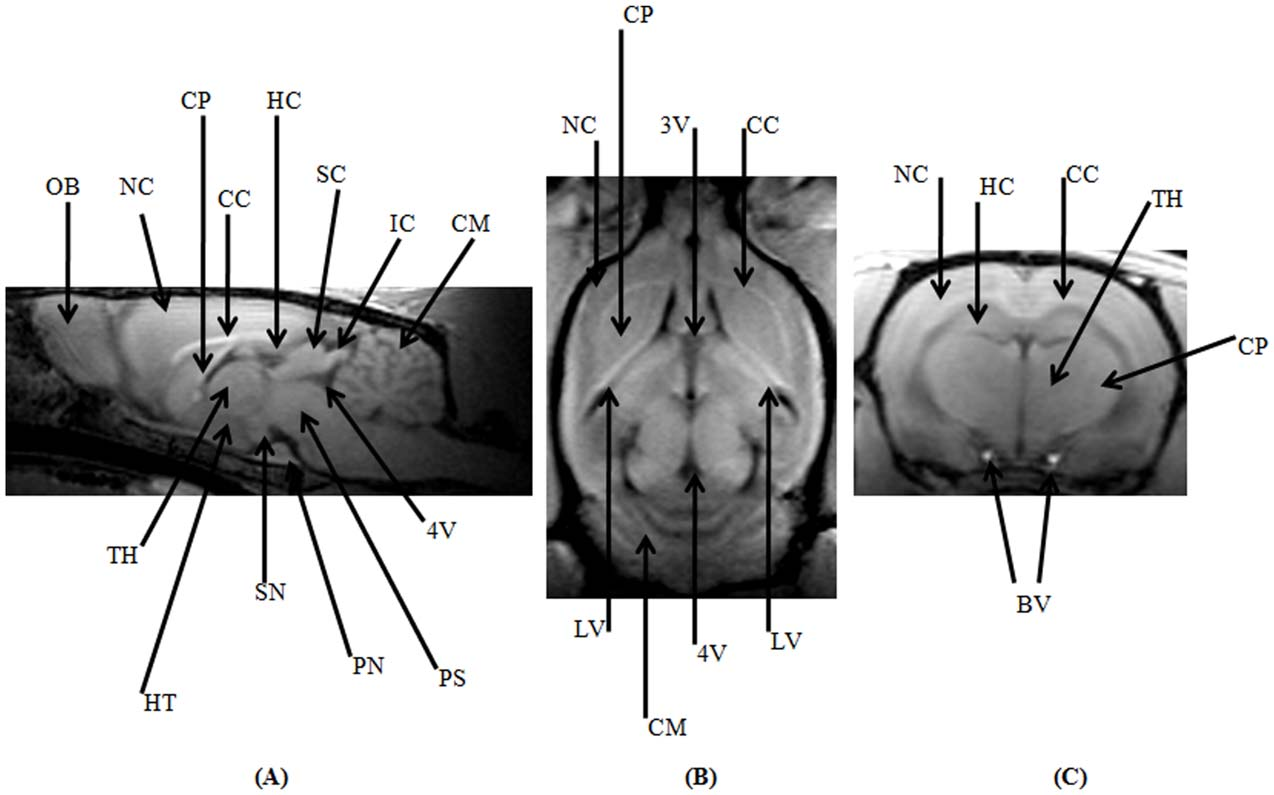
3D-MPRAGE data sets of a rat brain in three orthogonal planes. (A) Mid-sagittal plane, (B) Axial plane at the level of the eyes and, (C) Coronal plane. OB, olfactory bulb; NC, neocortex; CP, caudate putamen; CC, corpus callosum; HC, hippocampus; SC, superior colliculus; IC, inferior colliculus; CM, cerebellum; 4V, 4^th^ ventricle; PS, pons; PN, pontine nuclei; SN, substantia nigra; HT, hypothalamus; TH, thalamus; 3V, 3^rd^ ventricle; LV, lateral ventricle; BV, blood vessels(internal carotid arteries).

Representative images of an intact rat brain in mid-sagittal plane (Figure 4A), axial plane at the level of eyes (Figure 4B) and in the coronal plane (Figure 4C) are provided. The acquired images had an in-plane resolution of 200 µm with an ST of 1 mm. The 3D acquisition with 15 contiguous slices acquired with 4 averages consumed 18 minutes and 16 seconds. The T_1_-weighted MPRAGE sequence clearly represented the cerebrospinal fluid filled spaces as hypo-intense regions, the corpus callosum as a hyper-intense band separating the cortical and sub-cortical structures. The mid-sagittal section could clearly characterize a number of different anatomical regions like, the olfactory bulb, neocortex, caudate putamen, corpus callosum, cerebellum, 4^th^ ventricle, hippocampus, superior and inferior colliculus, pons, pontine nuclei, substantia nigra, hypothalamus and thalamic regions. The axial plane again portrayed the neocortex, corpus callosum, caudate putamen, cerebellum along with lateral, 3^rd^ and 4^th^ ventricles. The coronal section adequately represented many of the previously mentioned regions like the neocortex, corpus callosum, thalamus, caudate putamen along with the hippocampi. Two bright spots indicative of intact internal carotid arteries could also be clearly distinguished.

### 3. The rat model of cerebral ischemia

**Figure 5:**
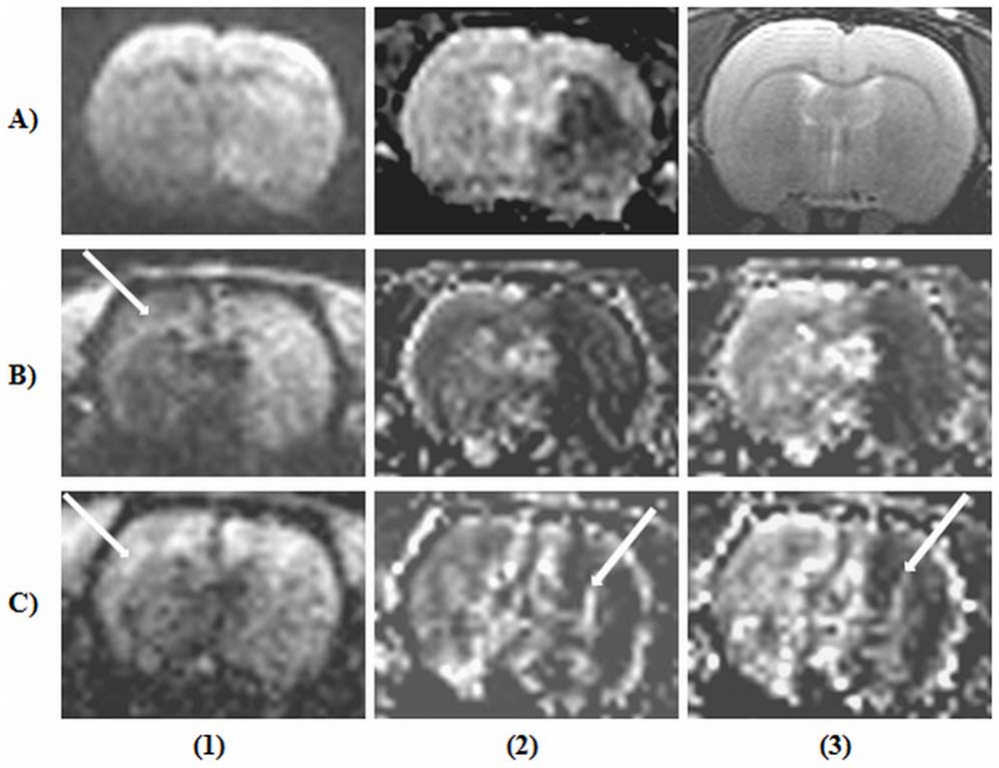
Representative images depicting diffusion and perfusion characteristics of an ischemic rat brain. Data sets acquired at 1 hour post-transient middle cerebral artery occlusion (tMCAO) includes, (A1) diffusion weighted echo planar image (b = 1000 s/mm^2^), (A2) Corresponding apparent diffusion co-efficient map, and (A3) T_2_-weighted turbo spin echo image. Row (B) represents perfusion echo planar images (PE-EPI) acquired with the parallel imaging reconstruction algorithm (RA), generalized auto-calibrating partially parallel acquisition at acceleration factor (AF) of 4. (B1) PE-EPI acquisition demonstrating altered perfusion characteristics following tMCAO, (B2) derived relative cerebral blood volume (rel-CBV) map and (B3) relative cerebral blood flow (rel-CBF) map. Row (C) represents PE-EPI images acquired with the RA, modified sensitivity encoding at AF 4. (C1) PE-EPI acquisition, (C2) rel-CBV map and (C3) rel-CBF map. Arrow marks on images (B1) and (C1) indicates differential sensitivity to first-pass gadolinium diethylene triaminepentaacetic acid and the arrows on (C2) and (C3) may probably indicate reconstruction related artefacts.

One hour post-tMCAO, sequels of diffusion and perfusion deficits, as confirmed by DW- and PE-EPI images along with a T_2_-weighted image is provided in Figure 5. Representative DW-EPI image (b = 1000 s/mm^2^) (Figure 5-A1) acquired with the PI RA GRAPPA and the corresponding ADC map (Figure 5-A2) clearly depict the ischemic area. The PE-EPI image (Figure 5-B1) acquired with GRAPPA demonstrates well defined regions of perfusion deficit. Derived perfusion parameters like, rel-CBV (Figure 5-B2), rel-CBF (Figure 5-B3), maps are in good agreement with the obtained DW-EPI and ADC images indicating compromised blood flow characteristics typical of this pathology. However, PE-EPI images (Figure 5-C1) acquired with mSENSE demonstrated reduced sensitivity to the first-pass of Gd-DTPA which can be clearly discerned, at the cortical region by a reduction in signal attenuation due to the passage of Gd-DTPA. The derived perfusion maps including rel-CBV (Figure 5-C2) and rel-CBF (Figure 5-C3) demonstrated altered rel-CBV and rel-CBF characteristics at the ischemic region which are not in total agreement with the GRAPPA derived maps. The acquired T_2_-TSE image without any noticeable changes is also provided (Figure 5-A3).

**Figure 6:**
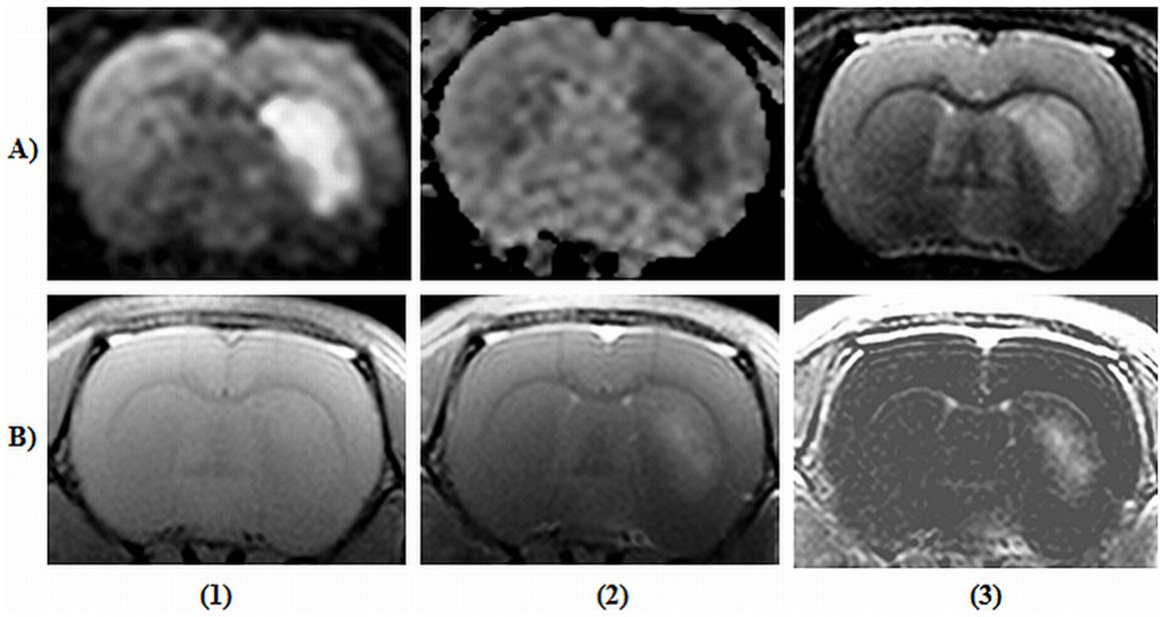
Representative images of a rat brain at 4 hours post-reperfusion. Following an hour of transient middle cerebral artery occlusion, animal was allowed to reperfuse for 4 hours before the following acquisitions were made. A1) Diffusion weighted echo planar image, A2) Apparent diffusion co-efficient map, A3) T_2_-weighted turbo spin echo image, B1) Pre-contrast T_1_-weighted spin echo image (T_1_-SE), B2) Post-contrast T_1_-SE image, B3) Subtraction image.

Representative images from another rat at 4 hours post-reperfusion are shown in Figure 6. Hyper intense ischemic lesions could be clearly delineated on DW-EPI and T_2_-TSE images (Figure 6-A1 & -A3), which co-localized with regions of reduced ADC (Figure 6-A2). Subtraction image (Figure 6-B3) derived from pre-and post-contrast T_1_-SE images (Figure 6-B1 & -B2) clearly depict regions of altered BBB permeability following ischemia-reperfusion injury. The acquired image characteristics are detailed in Table 6.

### 4. The rat model of intra-cerebral haemorrhage

**Figure 7:**
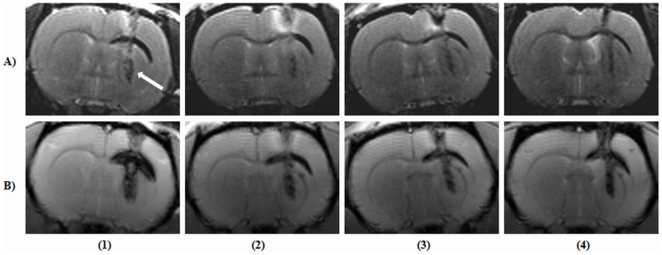
Image data sets obtained from a rodent brain following intra-cerebral haemorrhage. The study monitored up to one month includes, Row (A), T_2_-weighted turbo spin echo images. Row (B), T_2_*-weighted gradient recalled echo images. Column (1), images acquired within an hour of injury; (2) at 72 hours; (3) at 1 week; (4) at 1 month post-injury. The arrow indicates the bright rim surrounding the hematoma due to edema formation and/or extruded serum from the injected blood.

The longitudinal study performed at multiple time points with T_2_-TSE and T_2_^*^-GRE sequences duly detected blood and/or degraded blood products like haemosiderin during these time points (Figure 7). T_2_-TSE image within 1 hour of the injury portrayed the characteristic hyper-intense border along with hypo-intense representation of the location and extent of hematoma (Figure 7-A1). Susceptibility (T_2_^*^)-weighted images also spatially localized the hematoma throughout the intended duration of the study. The acquired image characteristics are as detailed in Table 6.

### 5. The rat model of cervical and thoracic spinal injuries

**Figure 8:**
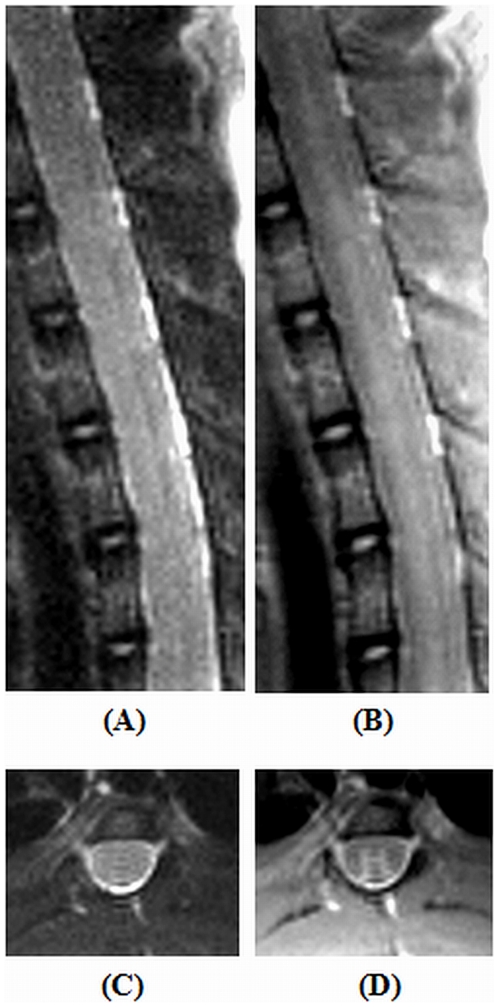
Images acquired from normal healthy rodent spine at the thoracic T(10) level. (A) and (C), T_2_-weighted turbo spin echo (TSE) images in sagittal and coronal planes. (B) and (D), proton density weighted-TSE images in sagittal and coronal planes.

The images obtained from the healthy intact rodent spine at the thoracic (T10) region characterized with T_2_- and PD- TSE sequences in both the sagittal and coronal planes are provided (Figure 8). No motion related artefacts were discernible with the images acquired in both planes. The T_2_-TSE coronal section (Figure 8C) could clearly delineate the spinal tissue from the bright encircling CSF, whereas, the PD-TSE image depicted the ‘H’ shaped gray matter (Figure 8D) from the surrounding white matter tissue.

**Figure 9:**
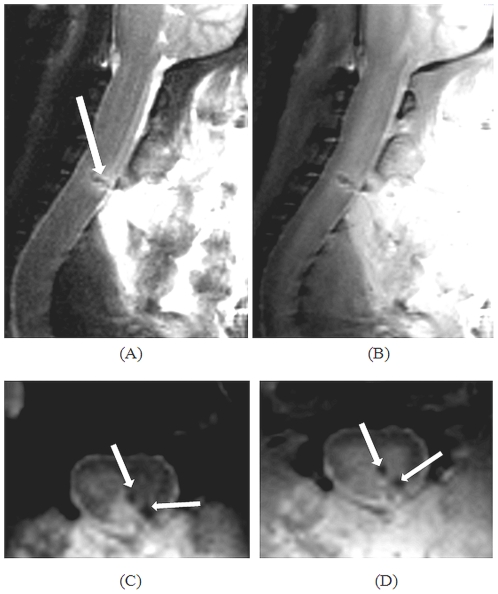
Tungsten wire knife induced cervical spinal lesion at 24-hours post-injury. (A) Sagittal T_2_-weighted turbo spin echo (T_2_-TSE) image. Arrow indicates the surrounding edema and/or serum (bright band) extruded from the blood; (B) Sagittal proton density (PD)-weighted TSE image; (C & D) Coronal T_2_-TSE and PD-TSE images. Arrow marks indicate localized hematoma formation.

Representative T_2_- and PD-TSE images in both coronal and sagittal planes of cervical lesion post 24 hours are provided (Figure 9). T_2_-TSE images clearly depicted the hyper-intense band attributed to edema/serum formation with hypo-intense spots due to hematoma (Figure 9-A&C). PD-TSE images also portrayed the same sequels albeit lower sensitivity but with added gray-white matter contrast (Figure 9-B&D).

**Figure 10:**
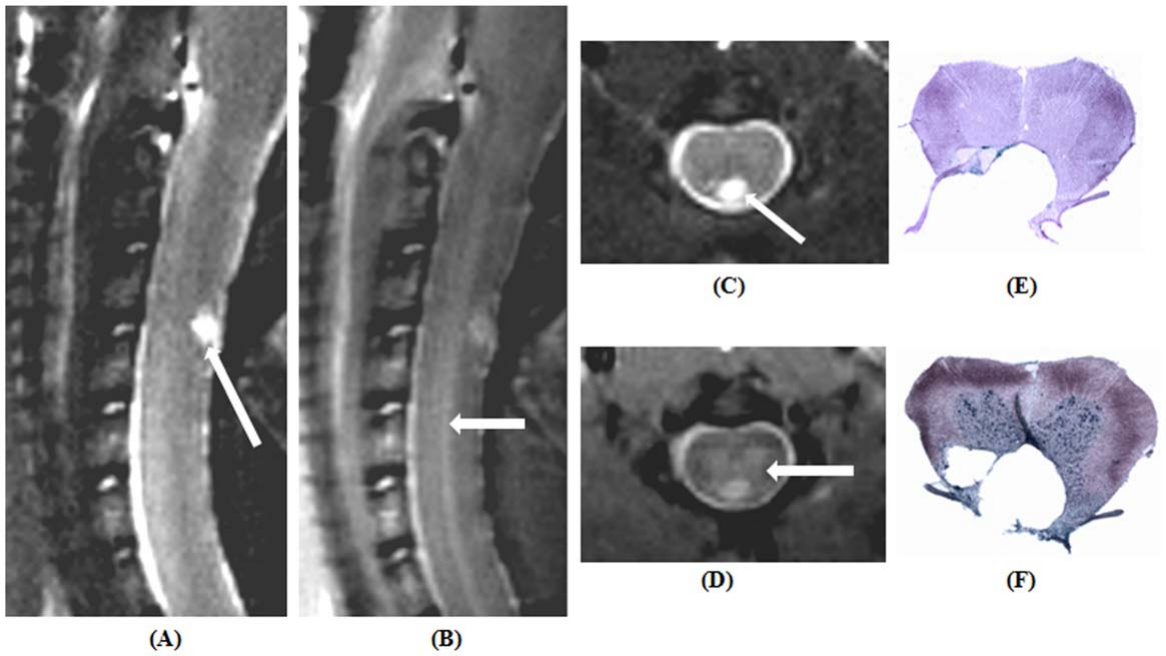
Tungsten wire knife induced cervical spinal lesion at 30-days post-injury. (A & C) Sagittal and coronal T_2_-weighted turbo spin echo (TSE) images. Arrows indicate fluid filled cyst as confirmed by Prussian blue and Nissl stained histological sections(E & F). (B & D) Sagittal and coronal proton density-weighted TSE images. Arrows indicates the visible gray matter tracts.

T_2_- and PD-TSE images in both coronal and sagittal planes from another animal 30 days post cervical injury along with their histology are provided (Figure 10). T_2_-TSE images clearly characterized cyst formation as confirmed by histological sections (Figure 10- A, C, E & F) whereas PD-TSE images portrayed injury along with a clear delineation of the gray-white matter structure confirmed by Nissl stained histology section (Figure 10- B, D & F).

**Figure 11:**
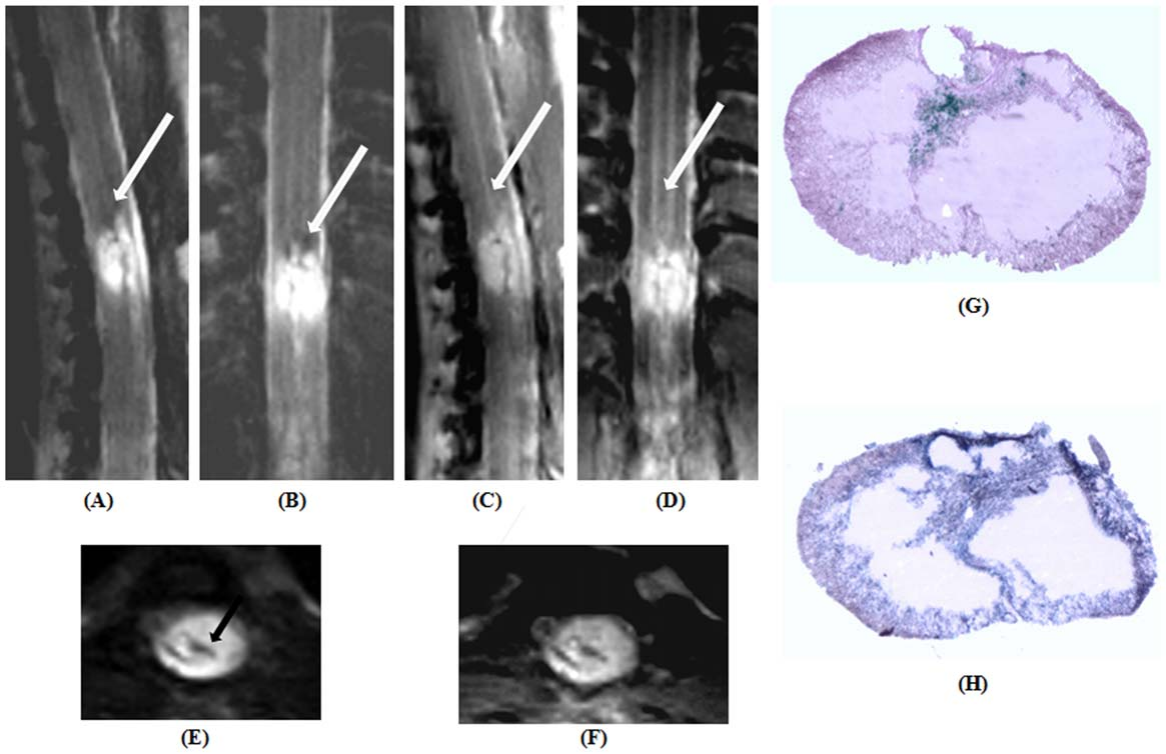
Representative images depicting spine contusion injury at the thoracic (T10) level post 30 days. (A, B & E) Sagittal, axial and coronal T_2_-weighted turbo spin echo (TSE) images. Arrow marks point to haemosiderin deposits, presence of which is confirmed by Prussian blue staining (G). (C, D & F) Sagittal, axial and coronal proton density-weighted TSE images. Arrow marks point to gray matter tracts, contrast of which is completely lost at the site of injury. (H) Nissl stained histological section confirms the complete loss gray matter tissue.

Images in multiple planes (sagittal, axial and coronal) from 30-day post-contusion lesion along with histological correlates are provided (Figure 11). T_2_-TSE images, irrespective of the acquisition planes sensitively captured haemosiderin and cyst formation as confirmed by Prussian blue staining (Figure 11-A, B, E & G). PD-TSE images demonstrated a complete loss of gray-white matter contrast at the site of injury as confirmed by Nissl stained histology and by the existed lower body paralysis of the animals (Figure 11-C, D, F & H). All image acquisition characteristics are provided in Table 3.

### 6. The mouse model of glioblastoma

**Figure 12:**
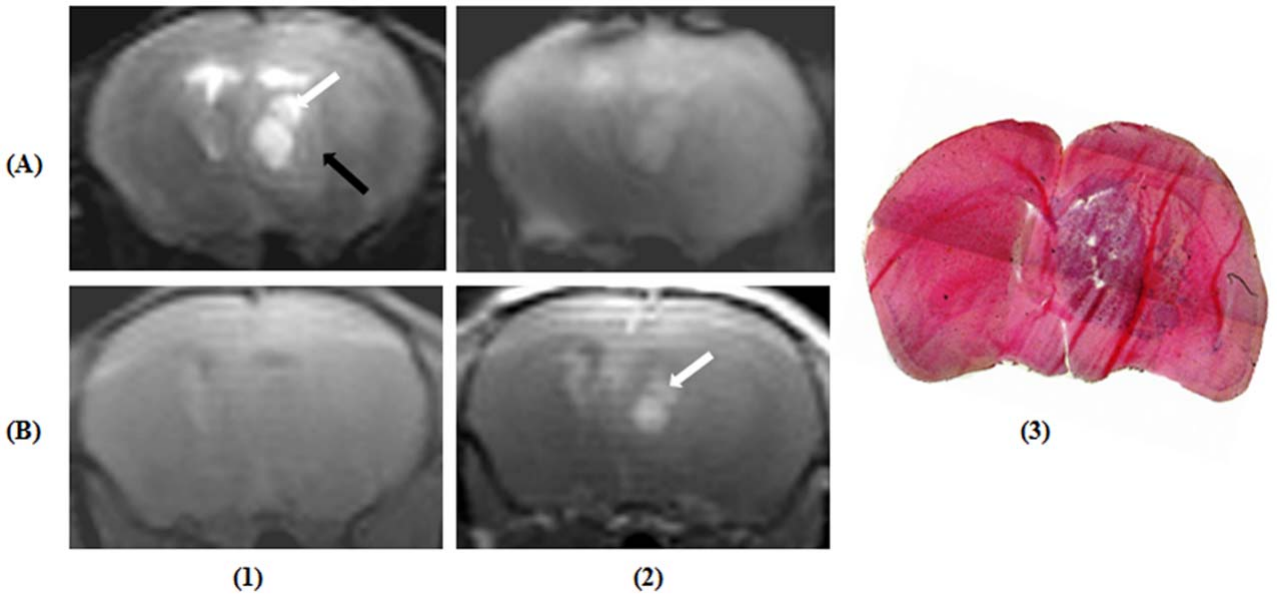
Representative images of a mouse brain with glioblastoma. (A1) T_2_-weighted turbo spin echo image. Bright arrow points to fluid filled necrotic core and dark arrow points to hyperintense regions attributed to invading tumour cells; (A2) Corresponding T_2_*-weighted gradient recalled echo image, (B1) Pre-contrast T_1_ -weighted spin echo (T_1_-SE) image, (B2) Post-contrast T_1_-SE image. Arrow indicate region of Gadolinium diethylenetriamine pentaacetic acid extravasation, (3) Hematoxyllin and Eosin stained tissue sections depicting the purple coloured tumourous tissue.

The MR signatures of HCSC induced murine xenograft GBM are given (Figure 12). T_2_-TSE image captured the fluid filled necrotic core of the tumour with surrounding hyper-intense regions indicative of invading tumour cells and these features closely correlates with the provided Hematoxyllin and Eosin stained histological section (Figure12-A1,3). Meanwhile, T_2_^*^-GRE image and the histology did not detect any haemorrhage which may co-exist with tumour (Figure 12-A2,3).As expected, post-contrast T_1_-SE image (Figure12-B2) clearly localizes Gd-DTPA extravasation to the necrotic core detected by T_2_-TSE image but not to the invading brain parenchyma. Image acquisition details are as provided in Table 6. The tumour volumes determined from T_2_-TSE images and from the histological study are detailed in Table 7. The Pearson correlation coefficient (r) was 0.9214 with significant correlation (P<0.0001).

**Table 7:** Comparison of tumour volumes determined by MRI and the histological study.

Animal ID	MRI tumour volume (mm^3^)	Histological tumour volume (mm^3^)
1	3.72	1.2
2	4.4	0.35
3	5.28	1.16
4	0.57	0.49
5	2.64	0.396
6	1.0	0.39
7	0.45	0.0
8	1.34	0.0
9	10.4	6.3
10	17	8.7
11	4.3	2.08
12	1.04	0.44
13	2.75	2.87
14	2.3	2.5
15	1.2	1.2

## Discussion

In this study we present a series of high-resolution MR images of small animal models with a variety of CNS pathologies. We performed quantitative relaxometry not only to optimize MR sequence parameters, but also to gain an understanding of these paradigms which is of paramount importance in research applications for at least two reasons: - A) Tissue relaxation characteristics are extremely sensitive to subtle changes like blood flow, tissue oxygenation and edema formation within minutes of the ictus [31]. B) Efficacy of therapeutic interventions could be better appreciated with the quantitative nature of such estimations. Since, no data is available with scanners of comparable field strength; these values may serve as a reference standard.

In our study, we have observed a significant difference between the T_2_^*^ values at the cortex and striatum in the brains of both rats and mice. Since T_2_^*^ values are influenced by a number of factors like field inhomogeneity, tissue oxygenation, blood flow and tissue distribution of paramagnetic substances like iron, a much more detailed study needs to be conducted to ascertain the causative factors behind this observation[32]. Studies have shown that a number of sub-cortical structures like the globus pallidus, ventral tegmentum and substantia nigra preferentially stores iron in rats which is particularly pronounced in elderly and female animals [33], [34]. Furthermore, studies in young mice have also demonstrated selective iron accumulation in the white matter tracts[35]. Even though such regional variations are a strong contender for the observed results especially in mice, we are not particularly ascertaining to this cause with the employed rats as they were relatively young at 6-8 weeks of age. Another factor that may be responsible is the very nature of the T_2_^*^-weighted GRE sequences. The T_2_^*^ value of the tissue has been determined by employing a range of TE values from 20–60 ms with a relatively low image matrix of 128×128 and the resultant in-plane resolution was 400 µ which is two times lower than the one employed for detecting cerebral haemorrhage. The acquisition of T_2_^*^ images at the relatively longer TE values of 50 and 60 coupled to the lower matrix size could have rendered the sequence too susceptible to field inhomogeneities rendering such a disparity between the two anatomic regions.

The employed MRI system, with a relatively smaller diameter of a spherical volume (DSV) at 220 mm had more magnetic field homogeneity (<0.1 parts per million (ppm) volume root mean square (Vrms)) compared to whole-body scanners which usually have a DSV of 400 mm and results in elevated <0.35 ppm Vrms. Such a small DSV also contributed to improved gradient performance with a doubled slew rate up to 400 mT/m/ms compared to 200 mT/m/ms of whole body scanners. The employed system also possessed a short magnet length of 1.25 m which was helpful particularly with small animal imaging with an easy access to the anesthetized animal for contrast/drug administration and animal monitoring. All the above-mentioned comparisons were performed against the whole body scanner, Siemens magnetom Trio (Siemens Health care Sector, Erlangen, Germany). To the best of our knowledge, this is the first small animal study performed on such a dedicated head scanner with customized phased array coils at 3 T.

Considering the implementation of PI strategies for DW- & PE-EPI sequences, DW-EPI image set without implementing any PI did not demonstrate gross geometric distortions and was readily comparable to those obtained with PI. This relative freedom from distortions probably arises from the adoption of a rectangular FoV and the resultant reduction of TE, from 197(for a square FoV) to 128(data not shown). The additional benefit of employing GRAPPA seems to stem out of two factors, namely, a concomitant increase in signal intensity as a result of a much shorter TE coupled to a reduced SD_diff_ which, in turn contributed to maximal resultant SNR when implemented at an AF of 3. Even though we could not directly compare our results with similar studies, the obtained data seems to be in good agreement with human studies carried out at identical field strength [36]. Even though, both GRAPPA and mSENSE demonstrated an increase in mean signal intensity with increase in AFs, GRAPPA provided a minimal SD_diff_ with an AF of 3 whereas SD_diff_ with mSENSE increased with higher AFs to the point where the image reconstruction program quit abruptly raising exceptions at the AF of 4, probably from unacceptable noise amplifications. Such distinct behaviour of both RAs could be due to factors like, the reconstruction methodologies followed, and the spatially varying geometry factor (g-factor), which in turn is again dependent on a plethora of factors like coil geometry, phase encoding direction and acceleration factor[25].

In addition, a comparison of both GRAPPA and mSENSE with PE-EPI sequence, in the absence of injected Gd-DTPA demonstrated identical results as in the case with DW-EPI by showing an increase in mean signal intensity which could be reasonably expected with a concomitant reduction in TE. However, the fact that mSENSE demonstrated consistently low SD_diff_ values is in contrast to its behaviour observed with DW-EPI sequences. Again, with an increase in AF from 3 to 4 both the RAs showed a reduction in the observed SD_diff_ values, which is also not in agreement with its early behaviour. We could not find similar studies either in animals or in humans where these RAs were compared in PE-EPI sequences making this a rather singular observation. Since, DW-EPI sequence happens to be a spin-echo EPI sequence with diffusion sensitizing gradients, whereas, PE-EPI sequence being a GRE-EPI sequence presenting two different environments might hold the key, amongst others as mentioned above, to the different behaviour of these two RAs with respect to variations in SD_diff_.

Even though, the present work does not attempt to perform MR phenotyping of rodent brains, the employed 3D-MPRAGE sequence delineated a number of anatomical regions. The employed inversion time of 900 ms was that of the striatum, since, we did not go for extensive relaxometric characterization of other anatomical regions. The obtained contrast, primarily between brain parenchyma and the cerebrospinal fluid may be due to the greater T_1_-contrast available with the short echo time, and the preparatory 180° pulse yielding an inversion recovery type of contrast. To the best of our knowledge, we could not find similar data at this field strength, except for one study employing spin-echo sequences for structural characterization of the rodent brain and was therefore unable to perform a cross comparison of results[17]. A number of works at 1.5 T has attempted to perform such anatomical characterizations and the added quality of the presented MPRAGE images can extend the scope of applications involving brain lesion detections, tissue grafting and enables morphometric studies[15], [37], [38].

### Imaging of cerebral ischemia

PE- and DW-EPI detected regions of perfusion deficits and accompanying ischemia at an early time point. DW-EPI images are acquired using relatively high ‘b’ values to ‘desensitize’ the sequence to fast moving protons such as those within blood vessels while sensitizing them to less mobile protons in the extra- and intra-cellular spaces[39]. Further, performing DW-EPI with more than three ‘b’ values also helped to minimize noise in the generated ADC maps [40]. Implementation of an adapted parallel imaging method has contributed enormously in improving the image quality of EPI sequences. The k-space based reconstruction, GRAPPA, generated alias free full FoV images without significant artefacts[23].

Gd-DTPA, the widely used paramagnetic lanthanide chelate, preferentially alters T_1_ and T_2_^*^ relaxation characteristics[41]. To accurately assess perfusion characteristics it is necessary to measure the drop in signal during passage of the bolus with high sensitivity to susceptibility changes at high temporal resolution. EPI sequences are primarily preferred due to their very short ATs. However, in EPI the whole MR signal during the read-out duration decays due to T_2_^*^ relaxation, which makes EPI sequences inherently sensitive to T_2_^*^ effects[42]. As a readout technique EPI could be combined with a number of excitation schemes and the GRE-EPI is superior to other EPI sequences with regards to signal-to-noise ratio and maximum signal reduction[42]. Since, they are susceptible to field inhomogeneities particularly at high fields; the enhanced field homogeneity offered by this dedicated head scanner has been complementary to the quality of the obtained perfusion images.

Even though mSENSE, provided superior results in terms of the obtained SNR and SD_diff_ with a normal healthy brain and in the absence of first pass Gd-DTPA, GRAPPA proved more sensitive to Gd-DTPA bolus passage and the reconstructed images were free from any artefacts. Once again, we could not directly compare our results to any previous studies either in animals or in humans. The most probable reason for the observed results with mSENSE could possibly be attributed to differences between distortions in EPI images and the obtained coil sensitivity maps[43]. Further, such differences could probably be augmented by the additional T_2_^*^ relaxation perturbations resulting from the first pass Gd-DTPA.

Following 3–4 hours of reperfusion, BBB permeability characteristics are altered resulting in increased water shift from the intra- to extra-vascular compartment accompanied by prolonged T_1_ and T_2_ relaxation effects [44], [45]. The fact that Gd-DTPA is of low molecular weight and its T_1_ interactions are only pronounced for short distances qualify it as a marker for assessing BBB integrity[46]. Since the T_1_-relaxivity (R_1_) shortens with field strength, contrast-enhanced imaging at higher field strengths could contribute to enhanced delineation of tissue volumes with compromised BBB permeability[47].

### Imaging of intracerebral haemorrhage

In the rodent model of hyper-acute ICH (≤1 hour), the extravasated blood should undergo de-oxygenation so that the heme iron in ferrous (Fe^2+^) form turns paramagnetic by virtue of its four unpaired electrons[48]. Oxygen extraction from blood is facilitated by the hypo-perfused, oxygen deprived surrounding tissue accompanied by rapid acidification causing the ‘Bohr effect’ to promote local oxygen dissociation from hemoglobin [49]. The observed band of high signal intensity on the T_2_-TSE images around the periphery of the hematoma portrays the distribution of extravasated serum from the clot or edema in adjacent brain parenchyma [49], [50]. T_2_^*^-GRE images may lead to a certain overestimation of the hematoma volume as they are also highly sensitive to magnetic field inhomogeneities induced by inherent susceptibility differences at the hematoma/tissue interface. Since high field strength and GRE techniques increase the sensitivity to magnetic susceptibility effects through different mechanisms, they are additive and produce marked signal loss when applied together[51].

### Imaging of spinal injuries

In-vivo MR studies of the rodent central nervous system comprising the spinal cord is particularly challenging owing to the relative small size of the cord which varies between 1–3 mm in diameter. Such small internal structures of rodents can easily overwhelm the limits of conventional clinical scanners. Such undertakings usually demand higher field strengths (up to 17.6 T) and implanted surface coils to maximize SNR while maintaining a smaller FoV [11],[52],[53],[54]. Even though impressive in terms of the obtained image quality, the 17.6 T system also had its drawbacks arising from a narrow bore, limiting air circulation resulting in anaesthetic vapour accumulation leading to death of animals[11]. Implanted surface coils also gives rise to unique problems, which, not only involve an invasive procedure, but also, such coils suffer from resonance frequency drifts over time, which is irreversible, resulting in loss of SNR and thereby rendering longitudinal studies almost impossible. Furthermore, since the cervical spinal cord is located relatively deep from the surface, the implantation procedure could cause widespread tissue damage[55].

Considering the rather harsh magnetic environment surrounding the cord, the commonly employed field strength for studying rodent spine happens to be at 7 T even though a number of workers have performed rodent spine imaging at the relatively lower field strength of 2.0 T allowing distinction of gray and white matter[56], estimating relaxation times[57] and diffusion characteristics[58], [59]. Almost all of these studies have been exclusively carried out with dedicated animal scanners using either external or implanted coils. Off late, a couple of studies have been performed with customized rat spine phased array coils, as they offer extended coverage, high SNR and PI capabilities[55], [60]. To the best of our knowledge, this is the only work that has been performed on a clinical scanner, with a 4-channel phased array coil, designed primarily to image the thoracic spine, was employed to image a wire knife cut injury at the cervical (C3) level. The employed sequence clearly detected edema and haemorrhage from the surrounding spinal cord parenchyma at the acute phase and de-lineated cyst formation at the extended time point of 30 days. The obtained IPR of 200 µ with an AT of 20–25 minutes is comparable to the only MRI study involving cervical spine obtained with a 7 T scanner employing a 3-channel phased array coil where the IPR was 175 µ at an AT of 15 minutes and 21 seconds[55].

MR imaging studies on spine contusion injury models have been carried out previously [11], [52], [61], [62]. Even though the present study is distinct by employing a clinical scanner and a phased array coil system, we were able to clearly detect gray-white matter differentiation and de-lineation of cyst formation and haemosiderin deposits at the extended time point following 30 days from the injury. Consistent with previous studies, MR images consistently depicted hypo intensities rostral to the contusion injury at the thoracic level, which was confirmed as haemosiderin deposits due to trauma induced haemorrhage by Prussian blue staining [52]. From our results, most of the hypo intense signals arise from the gray matter, which was also observed by another study where the haemorrhage was mainly confined to the gray matter at the acute time point[63]. However, at least one study at the higher field strength of 17.6 T demonstrated signal changes even in the white matter[11]. Such high field strength and its dependent changes in relaxation times and spatial resolution could have enabled the visualization of relatively discrete haemosiderin deposits in white matter tracts[64]. Another aspect is that, this study was conducted 58 days post injury and that is almost double the time allowed for our study which was restricted to 30 days. It remains to be determined whether, an extended time period allows for such an appearance.

The loss of gray-white matter contrast is the hallmark of spinal contusion injuries, which is invariably associated with lower body paralysis. An early study had noted a return of the gray matter, indicated by added contrast between the gray and white matter at late time points from 3–8 weeks post trauma and the authors attribute this to neuronal recovery [52]. PD-TSE sequences are of paramount importance in spinal cord injury as it is the only one that can clearly delineate the gray and white matter tracts. The return of gray-white matter contrast in images acquired by PD-TSE sequences therefore serves as a surrogate marker for clinical improvement.

### Imaging of mouse brain tumour

The employed phased array coil assembly has been designed to image rodent brains with volumes of 600±10.6 mm^3^ [65]. The mouse brain (269.16±2.2 mm^3^) being much smaller has been preferentially studied at higher field strengths even though murine GBM have also been characterized using clinical scanners [30], [66], [67], [68]. GBM is characterized by high angiogenic activity accompanied by loss of permeability characteristics at BTB and/or BBB structures leading to lethal edema formation[69]. The TE value of the T_2_-TSE sequence, determined by tissue relaxometry was instrumental to the sensitive representation of tissue hyper-intensity surrounding the necrotic core indicative of tumour proliferation and helped to differentiate between tumour necrosis and brain parenchyma invaded by tumour cells. In addition, the scans obtained showed a remarkably high correlation to the histological findings underscoring the quality of the established sequence parameters.

Taken together, it could be concluded that the lack of a dedicated high-field scanner shouldn't be a deterrent to carry out small animal imaging studies. This study demonstrates the importance of a certain degree of user-end customisations, including the dedicated RF coil system along with careful optimization of sequence protocols which can dramatically improve the quality of images. Further work would extend the prospects of such an endeavour and will broaden the required armamentarium for neuroscience research.
